# Feasibility of self-collection of fecal specimens by randomly sampled women for health-related studies of the gut microbiome

**DOI:** 10.1186/1756-0500-7-204

**Published:** 2014-04-01

**Authors:** Heather Spencer Feigelson, Kimberly Bischoff, Mary-Anne E Ardini, Jacques Ravel, Mitchell H Gail, Roberto Flores, James J Goedert

**Affiliations:** 1 Institute for Health Research, Legacy Highlands, Suite 300, Kaiser Permanente Colorado, P.O. Box 378066, Denver, CO, USA; 2 RTI International, Multisite Epidemiology and Statistics, Research Triangle Park, NC, USA; 3 Institute of Genome Sciences, University of Maryland School of Medicine, Baltimore, MD, USA; 4 Biostatistics Branch, Division of Cancer Epidemiology and Genetics, National Cancer Institute, Rockville, MD, USA; 5 Infections and Immunoepidemiology Branch, Division of Cancer Epidemiology and Genetics, National Cancer Institute, Rockville, MD, USA; 6 Cancer Prevention Fellowship Program, National Cancer Institute, Rockville, MD, USA

**Keywords:** Study design, Microbiome, Breast cancer

## Abstract

**Background:**

The field of microbiome research is growing rapidly. We developed a method for self-collection of fecal specimens that can be used in population-based studies of the gut microbiome. We conducted a pilot study to test the feasibility of our methods among a random sample of healthy, postmenopausal women who are members of Kaiser Permanente Colorado (KPCO). We aimed to collect questionnaire data, fecal and urine specimens from 60 women, aged 55–69, who recently had a normal screening mammogram. We designed the study such that all questionnaire data and specimens could be collected at home.

**Results:**

We mailed an invitation packet, consent form and opt-out postcard to 300 women, then recruited by telephone women who did not opt-out. Verbally consented women were mailed an enrollment package including a risk factor questionnaire, link to an online diet questionnaire, specimen collection kit, and instructions for collecting stool and urine. Specimens were shipped overnight to the biorepository. Of the 300 women mailed an invitation packet, 58 (19%) returned the opt-out postcard. Up to 3 attempts were made to telephone the remaining women, of whom 130 (43%) could not be contacted, 23 (8%) refused, and 12 (4%) were ineligible. Enrollment packages were mailed to 77 women, of whom 59 returned the risk factor questionnaire and specimens. We found no statistically significant differences between enrolled women and those who refused participation or could not be contacted.

**Conclusions:**

We demonstrated that a representative sample of women can be successfully recruited for a gut microbiome study; however, significant personal contact and carefully timed follow-up from the study personnel are required. The methods employed by our study could successfully be applied to analytic studies of a wide range of clinical conditions that have been postulated to be influenced by the gut microbial population.

## Background

After menopause, breast cancer occurs at greater frequency in women who have high levels of estrogens [1,2]. Postmenopausal estradiol level is linearly related to body mass index (BMI), reduced with surgical menopause (bilateral ovariectomy), and elevated among current cigarette smokers and heavy consumers of alcohol [3]. Differences in estrogen levels throughout the body may also reflect differences in estrogen metabolism and elimination, especially in feces.

Metabolism of estrogens occurs predominantly in the liver, including hydroxylation and conjugation (glucuronidation, sulfation or O-methylation) [4]. Conjugated estrogens may be excreted in the urine or bile. From the bile, conjugated estrogens enter the gastrointestinal tract and interact with gut bacteria, where highly variable concentrations of microbial β-glucuronidase and other enzymes de-conjugate them, following which these biologically active hormones are reabsorbed through the mucosa and enter the circulation through the portal vein. It is estimated that 80% of excreted estrogens in bile are reabsorbed [5,6]. We hypothesize that systemic estrogen levels, and thus risk for postmenopausal breast cancer and perhaps other conditions, may reflect differences in the composition or functions of the gut microbial population, which can be measured from a single fecal sample.

An initial assessment of the bacterial population of the distal gut can be obtained by DNA that is extracted from feces [7]. However, collection of fecal samples for epidemiologic research is challenging. In addition to logistical complications, there is the potential for participation bias related to socio-demographics, perceived risk, or other motivations. Here we describe a study to determine the feasibility of successfully identifying and enrolling a representative sample of healthy postmenopausal women and collecting from them breast cancer risk factor data, urine and stool specimens using a mailed collection kit. Estrogen and estrogen metabolites could be measured from the urine samples, and fecal microbiota could be characterized from the stool sample. The primary aims of the Breast and Colon Health (BRanCH) feasibility study were to pilot test study materials and specimen collection among postmenopausal women from the general population of Kaiser Permanente Colorado (KPCO) members, to estimate response rates, and to identify differences between participants and non-participants that may need to be considered in subsequent studies.

## Methods

The study protocol was developed by collaborators at the National Cancer Institute (NCI), Kaiser Permanente Colorado (KPCO), the Institute for Genome Sciences at the University of Maryland School of Medicine (IGS), and RTI International (RTI). The study protocol and all study materials were approved by the Institutional Review Boards at each institution.

We defined eligibility criteria as current female KPCO members, aged 55–69, who had received a normal screening mammogram within the past 6–8 weeks. The virtual data warehouse (VDW) at KPCO was used to identify eligible women. The VDW is a standardized database of inpatient and outpatient diagnoses, procedures, laboratory results, and medications derived from the electronic medical record (EMR) [8]. Using the VDW, we excluded women with prescription use or conditions that could strongly impact either the normal gut microbial population or circulating hormone levels including the following: any history of prior cancer (other than non-melanoma skin cancer), inflammatory bowel disease or diverticulitis, gastric banding or by-pass surgery; history of other gastric or intestinal surgery (such as appendicitis) within the previous 6 months, any progesterone or estrogen hormone prescription within the previous 12 months, and antibiotic prescription within the previous 6 months.

Each month, approximately 2,400 women meeting the criteria above receive notification by mail that they have had a normal screening mammogram. From this population, we randomly sampled women to receive a recruitment package containing a brochure, introductory letter, consent document, and opt-out postcard. Two weeks later, unless the opt-out postcard had been mailed back, a KPCO research assistant, using a telephone script, called the potential participant to screen for conditions that were cause for exclusion (for example, recent antibiotic or hormone use or exclusionary conditions that were not recorded in the medical record) and to review the BRanCH study procedures and consent document. This was a rolling recruitment linked to date of screening mammogram, so that letters were mailed within six weeks of mammography. Recruitment packages were sent in four batches, approximately 1 month apart.

The first two batches included 100 women each, the second two batches included 50 women each, for a total of 300 potentially eligible randomly sampled women.

Participants who provided verbal consent were sent a second package, including a self-administered cancer risk-factor questionnaire, instructions for completing the on-line Block Brief Food Frequency Questionnaire (FFQ) [9], a specimen collection kit, illustrated instructions for collecting a single urine sample and four aliquots from a single fecal sample, and materials to ship the specimens overnight to the biorepository.

### Questionnaires, demographic, and clinical data

The self-administered risk factor survey included 77 questions on demographics, smoking, alcohol consumption, physical activity, body habitus, dietary restrictions, consumption of yogurt or probiotics, recent consumption of high-nitrite foods, family history of cancer, bowel habits as well as reproductive and menstrual history. A pre-addressed, postage paid envelope was provided for return of the completed form to RTI for double key data entry.

The on-line FFQ asks about usual dietary intake from 70 food items. It was designed to provide estimates of usual and customary dietary intake. The food list for this questionnaire was developed from the NHANES III dietary recall data. The nutrient database was developed from the USDA Nutrient Database for Standard Reference. Individual portion size is asked, and pictures are provided ( http://www.nutritionquest.com/). When finished, the participant could print out a free nutrition report based on her answers. The on-line questionnaire was considered optional because we did not know how many women had internet access and were comfortable completing data on-line.

Using the VDW, KPCO extracted information from the EMR, including age, ethnicity, geocode (as a surrogate for socioeconomic status), length of KPCO enrollment, potentially relevant medications and diseases, BMI, radiology codes for most recent mammogram, and history of breast biopsies.

### Specimens

The participant received printed illustrated instructions, a plain cardboard shipping box, a stool catching pouch, a Styrofoam box with a custom foam insert that held four 10 mL screw top Sarstedt tubes, a 120 mL screw-top container for urine (without preservative), and two gel packs to be frozen upon receipt of the kit. The specimen collection tubes were pre-labeled with only a unique code number, no personal identifiers. On the self-selected morning, excluding Friday through Sunday, the participant collected the urine and attached the stool catching pouch to the toilet seat. Defecating normally, the participant used the clean scoop attached to the tube lid to collect an aliquot, sealed the tube by screwing on the lid, and moved on to the next tube, collecting four aliquots in total. Two tubes were pre-loaded with 5 mL RNAlater (Qiagen) and two with 5 mL sterile phosphate buffered saline (PBS). The tubes were then secured in the foam insert for shipping and frozen gel packs were added to the shipping box. The participant called to arrange for package pick-up and overnight delivery to the NCI biorepository. Following receipt of the specimens at the repository, participants were sent a gift card with a small monetary value to convey our appreciation for their time and effort. Specimens were sub-aliquotted and frozen for future analysis.

### Statistical analysis

Our results are primarily descriptive. Enrollment was defined as receipt of fecal and urine specimens, as well as the self-administered risk factor survey. We compared enrolled women to those who refused and to those who could not be reached by telephone using an unpaired t-test for continuous variables (age, years of membership at KPCO, and BMI) and chi-square or Fisher’s exact tests for categorical variables (race/ethnicity as Non-Hispanic White vs. other, attended college (yes/no), and history of breast biopsy (yes/no).

## Results

**Figure 1:**
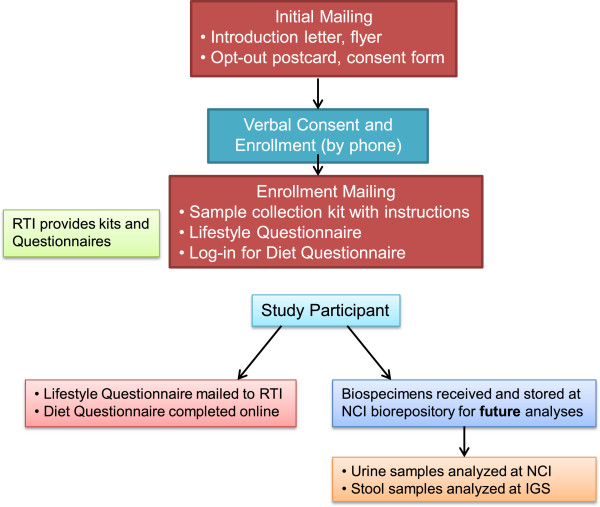
**BRanCH Study Overview showing roles of collaborative partners and enrollment activities.** NCI: National Cancer Institute; IGS: Institute for Genome Sciences at the University of Maryland School of Medicine; RTI: RTI International.

**Figure 2:**
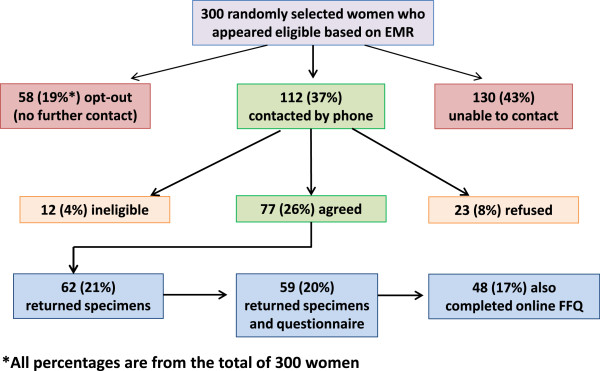
Enrollment cascade and response rate.

Over the course of six months, we intended to contact up to 300 women, with a goal to obtain specimens from at least 60 women. Participant recruitment occurred at KPCO, data and biospecimen processing was managed by RTI, urine estrogens and fecal specimens were stored at the NCI biorepository (Frederick, MD) for future analyses (Figure 1). The enrollment cascade is shown in Figure 2. Of the 300 women who were mailed an invitation packet, 58 (19%) returned the "opt-out" postcard and were not contacted further. A KPCO research assistant made up to 3 attempts to contact the remaining women by phone, of whom 130 (43%) could not be contacted, 23 (8%) refused, and 12 (4%) were found to be ineligible. Of those found to be ineligible, 2 had a history of cancer; 1 was pre-menopausal; 3 were taking estrogen; 3 had a history of gastrointestinal disease and 3 had recent antibiotic use.

We mailed enrollment materials to 77 (26%) women who agreed to participate. Of these, 62 women (21% of the randomly selected population) returned specimens. Fifty-nine (20%) women were classified as enrolled, returning both the risk factor survey and specimens. Forty-eight (17%) women completed the on-line food frequency questionnaire (FFQ), in addition to providing specimens and completing the risk factor survey. Of the 14 who did not complete the FFQ, eleven indicated they did not have internet access. Participants spent 1–2 hours collecting specimens, packaging them for shipment, and completing the risk-factor questionnaire. The FFQ took 30 minutes to complete on average.

We mailed 100 letters for each of the first two batches of the study. On average, the research assistant made 3 phone calls on different days and spent 25 minutes to verify eligibility, consent and enroll participants. In an attempt to reach potential participants more quickly and hopefully improve participation, we mailed 50 letters in the second two batches. Enrollment by batch was: 15% batch 1, 21% batch 2, 20% batch 3, and 26% batch 4. In addition to reducing batch 4 to 50 letters, we accelerated both initial and follow-up phone calls with a second research assistant.

The age range of enrolled women was 55–69 years (mean age 59.9 years) and the mean length of enrollment in KPCO was 12 years. Fifty (84.7%) of the 59 enrolled women were non-Hispanic white, 3 (5.1%) were African-American, 1 (1.7%) was Hispanic, and race/ethnicity was missing for 5 (8.5%) women. The majority (70.1%) of women who enrolled had attended college. Table 1 shows the characteristics of enrolled women who returned specimens and risk factor surveys (n = 59) compared to those who refused, either by returning the opt-out postcard or by phone (n = 81), and women we were unable to reach by phone (n = 130). We observed no statistically significant differences between the three groups of enrolled, refused, or unable to contact. However, enrolled women tended to be non-Hispanic White (84.7%, 67.9%, and 71.5%, respectively, p = 0.07), and were non-significantly more likely to have a history of breast biopsy (6.8%, 2.5% and 5.4%, respectively, p = 0.49) than the other two groups.

**Table 1:** Characteristics of the study population

	**Enrolled**^**1 **^**(n = 59)**	**Refused**^**2 **^**(n = 81)**	**Unable to contact (n = 130)**	**p-value**^**3**^
Mean age (range)	59.9 (55–69)	60.7 (55–69)	59.1 (55–68)	0.54
Non-hispanic white^4^	84.7%	67.9%	71.5%	0.07
Mean years in KPCO	12.0	12.4	11.4	0.72
Attended College	70.1%	67.1%	66.5%	0.41
Mean BMI	28.0	26.3	29.2	0.81
History of breast biopsy	6.8%	2.5%	5.4%	0.49

^1^Enrolled and returned specimens and questionnaire.

^2^Opt-out or refused over the phone.

^3^p-value compares enrolled to all others.

^4^Self reported race/ethnicity.

The risk-factor survey included four items on the ease or difficulty of various components of the study. We also allowed space for open-ended response. Of the 59 returned questionnaires, 24 had at least one comment or suggestion. The enrolled women had few negative comments or difficulties collecting the samples; only 2 found specimen collection more difficult than they expected. The biggest challenges mentioned for completing the study as requested was the restriction to collect stool samples only on Monday through Thursday mornings, and difficulty scheduling pick-up with the overnight courier.

## Discussion

These data demonstrate that an apparently representative sample of women from a general population can be successfully recruited for a gut microbiome study that involves collection of a fecal sample. Enrolled women were generally representative of the overall sample, although White non-Hispanic women tended to participate at a higher rate. While the enrollment rate calculated as a percentage of the number of women identified as potentially eligible was low (20%), it is not unexpected given the participant burden. Our 20% response rate is comparable to those reported by other large-scale studies that have enrolled healthy participants [10-12], but lower than is typically reported for case–control studies [13]. Further, once women initially agreed to participate via phone, they complied at 80% after receiving the kit and understanding the complexity of the task at hand. Few women listed specific problems with the specimen collection but did spend over one hour collecting specimens, packaging them for shipment, and completing the surveys. This amount of time commitment requires motivated individuals. Fortunately, our study engendered this type of motivation by clearly illustrating the importance of the goal and the steps required to comply and by providing reminders and other aids to the willing participant such as easy to use materials.

The success of this type of study requires significant personal contact and carefully timed follow-up. The KPCO research assistant made several attempts to contact potential participants, left messages when possible, and usually scheduled a convenient time to call back to discuss the study. We had better response from the last batch of mailed letters when women were called more promptly after receiving the letter of invitation, and follow-up calls were made after the collection kits were mailed to check for questions.

The KPCO Virtual Data Warehouse (VDW) facilitated identification of eligible women for this study. Women were identified from electronic mammography codes indicating a normal screening mammogram within weeks of receiving the test. We used the medical record data to apply our exclusion criteria prior to contacting any women for participation. At the time of the first phone call, the research assistant verified eligibility, including recent initiation of antibiotics or hormone therapy, and use of over the counter or herbal hormone therapies that would not be captured in the electronic medical record (EMR). Because of the extensive data available in the VDW, only 4% of the women contacted by phone were ineligible.

In-home sample collection offers both conveniences and challenges. To ensure the samples arrived at the NCI repository during a standard Monday through Friday working week, we requested that samples be collected and shipped on specified days of the week (Monday – Thursday). Although this did not greatly impact compliance from women who agreed to participate, it was inconvenient for many women whose work or personal schedules did not fit easily into this sample collection time frame. Some women also reported difficulty arranging a pick-up at their home. To address these issues of timing in future studies, we plan to provide participants with a sealed container that can be stored in the home freezer. The participant will put the urine and fecal specimens in the sealed container, immediately put this in her freezer, and then arrange a convenient time for the frozen samples to be picked-up by study staff equipped with a dry ice chest. This will allow for specimen collection to be done at the participant’s convenience, at any time of day, any day of the week. Also, this will ensure sample integrity, as the samples can be batched and shipped frozen to the repository.

## Conclusions

Although collection of fecal specimens from the general population is challenging, our results show that it is feasible. Our enrolled population was similar to those who refused or could not be contacted. However, our sample had limited power to identify differences between groups, thus generalization of our results to the larger population requires caution. Participation requires both motivated subjects and a significant amount of phone contact from the study staff. The methods employed by our study could successfully be applied to analytic studies of a wide range of clinical conditions that have been postulated to be influenced by the gut microbial population [14,15].

## Competing interests

The authors have no competing interests to declare in relation to this manuscript.

## Authors’ contributions

HSF and JJG conceived the study and acquired funding for the research. All authors participated in the design of the study, development of the protocol and data collection materials and sample collection kits; KB managed the study at KPCO. MEA managed the data at RTI; All authors read and approved the final manuscript.
